# Editorial: Osteoarticular-immunological interplay in response to disease and therapy

**DOI:** 10.3389/fimmu.2022.1052196

**Published:** 2022-10-11

**Authors:** Lisa Deloch, Maria Filkova, Michal Tomcik

**Affiliations:** ^1^ Department of Radiation Oncology, Universitätsklinikum Erlangen, Friedrich-Alexander-Universität Erlangen-Nürnberg, Erlangen, Germany; ^2^ Translational Radiobiology, Department of Radiation Oncology, Universitätsklinikum Erlangen, Friedrich-Alexander-Universität Erlangen-Nürnberg, Erlangen, Germany; ^3^ Institute of Rheumatology, Department of Rheumatology, 1st Faculty of Medicine, Charles University, Prague, Czechia

**Keywords:** bone, cartilage, rheumatoid arthritis, osteoarthritis, radiotherapy, osteoclast, T cell, B cell

Osteoimmunology is defined as an interdisciplinary research area that connects immunology and osteology (1–3). In articular diseases such as rheumatoid arthritis (RA) and osteoarthritis (OA) cells in the synovium, such as synovial fibroblasts, interact with immune cells and the bone to perpetuate the pathogenic mechanisms (4, 5). These cells, therefore, represent additional vital targets to improve therapeutic efficacy.

While the involvement of the immune system has been generally accepted in RA (5–7), this was only recently recognized in OA, where increasing attention has been directed towards systemic inflammation (8), also stressing the importance of the interplay between the synovium, cartilage, and subchondral bone (9). While a plethora of therapeutic modalities are available for the treatment of degenerative diseases of the joint, timely intervention is of the essence to prevent irreversible damage to the bone and joints. A concise understanding of the osteoarticular-immunological interplay is thus essential in providing targeted, additive, and comprehensive treatment options for these patients.

One of the additive treatment options is the so-called low-dose radiotherapy (LDRT) with low doses of ionizing radiation (mostly X-rays) that has analgesic and anti-inflammatory properties (10); however, many of the underlying mechanisms are not completely understood. Donaubauer et al. assessed immune modulations in patients with chronic degenerative and inflammatory diseases in the IMMO-LDRT01 trial (NCT02653079) and found a significant improvement in pain levels, modulations of circulating immune cells (e.g., a decrease in B cells) alongside a reduction in their activation status. Additionally, Eckert et al. examined the effects of LDRT on osteoclast differentiation and bone resorption in the same cohort and found that circulating monocytes, which were *ex vivo* differentiated into osteoclasts, showed lower numbers of differentiated osteoclasts while apoptotic levels remained low. This decrease in osteoclasts could potentially be due to reduced numbers of nuclei, suggesting an impaired fusion of pre-osteoclasts and a decrease in the Nuclear Factor of Activated T Cells 1 (NFATc1). Likewise, reduced bone resorption was also found in the LDRT group.

One of the biggest drawbacks of LDRT is the lack of placebo-controlled studies, as stated by Weissmann et al. They chose a translational approach and analyzed patient data as well as a pre-clinical model to identify parameters that could be implicated in future patient studies. They found that age and the localization of the affected joint are essential factors in LDRT. Furthermore, pre-clinical data revealed that the potential analgesic effects observed in patients could potentially be determined by an anti-inflammatory response (e.g., a reduction in serum interleukin (IL)-17A levels and a shift from CD8+ to CD4+ T cells in the bone marrow).

Liu et al. used a computational approach to identify potential new therapeutic targets in OA. They looked into five GEO datasets containing normal healthy and OA synovial tissues and identified six genes to be used as potential biomarkers for OA. In particular, they identified Stimulator of Chondrogenesis 1 (SCRG1), a protein-coding gene, as a diagnostic marker of OA, which is up-regulated in OA synovial tissue by hsa-miR-363-3p and regulates immune-related pathways, thus may become a potential new therapeutic target in OA.

To further elucidate the molecular mechanisms, Navrátilová et al. examined the role of B cell associated IL-40 in RA. They found IL-40 to be overexpressed in RA synovial tissue in contrast to the synovial tissue of OA patients, and higher IL-40 levels in the synovial fluid and serum of RA patients. The serum IL-40 levels in RA patients decreased following B cell-depleting therapy. Moreover, both local and systemic levels of IL-40 were associated with levels of autoantibodies, and levels of IL-40 in synovial fluid positively correlated with chemokines and markers of NETosis that unraveled a potential association of IL-40 with neutrophils. Relatedly, IL-40 induced the secretion of chemokines and MMP-13 by synovial fibroblasts *in vitro*.

Another study by Singh et al. demonstrated that transcription factor ETS Proto-Oncogene 2 (Ets2) induced osteoclast-like alterations in RA synovial fibroblasts (RASF). RASF are essential contributors to inflammation and bone destruction in RA *via* a plethora of cytokines and chemokines, including IL-6. While IL-6 contributes to bone loss, the molecular mechanisms of RASF/IL-6-induced bone loss are not completely understood. They found that Ets2 has a fundamental role in IL-6/IL-6 receptor signaling in RASF and thus modulates RASF heterogeneity and bone loss by transforming RASF into osteoclast-like cells.

Moreover, the interplay between Foxp3^+^ T cells and osteoclasts was the research subject of Dohnke et al. They carried out an extensive characterization of Switching B Cell Complex Subunit SWAP-70 controlled F-actin dynamics and found SWAP70 to be a potential new member of the Foxp3-dependent canonical Treg cell signature. SWAP70-deficient mice failed to efficiently suppress CTLA4/CD80/CD86 mediated bone homeostasis by impairing osteoclastogenesis as well as osteoclast function and thus representing a functional defect of Treg mediated suppressor function.

Similarly, due to a lack of understanding of the molecular processes, the etiology of degenerative joint diseases remains elusive. Ma et al. demonstrated that patients with periodontitis showed a higher risk of developing OA and even severe OA that required knee or hip replacement. Interestingly, OA patients also had a higher risk of developing periodontitis. They suggest that periodontitis following OA might be due to poor oral hygiene as a result of the lack of activity and patient education. A potential explanation for OA following periodontitis could include common underlying inflammatory conditions making certain individuals susceptible to both diseases.

The studies that have been reviewed in this Research Topic show the diversity of osteoarticular-immunological mechanisms as seen in disease and therapy. While more and more factors contributing to this interplay are being unraveled, additional research is needed to connect these findings and to further elucidate the underlying molecular mechanisms. Further research will ultimately facilitate an extensive understanding of the disease onset and progression, and consequently the identification of therapeutic targets for more efficient and personalized treatment options.

## Author contributions

All authors listed have made a substantial, direct, and intellectual contribution to the work and approved it for publication.

## Funding

Funding for LD by the Bundesministerium für Bildung und Forschung (BMBF; TOGETHER 02NUK073; GREWIS, 02NUK017G and GREWIS-alpha, 02NUK050E), for MF and MT by the Ministry of Health Czech Republic (023728, NU22-05-00226).

## Conflict of interest

The authors declare that the research was conducted in the absence of any commercial or financial relationships that could be construed as a potential conflict of interest.

## Publisher’s note

All claims expressed in this article are solely those of the authors and do not necessarily represent those of their affiliated organizations, or those of the publisher, the editors and the reviewers. Any product that may be evaluated in this article, or claim that may be made by its manufacturer, is not guaranteed or endorsed by the publisher.
